# Gels That Serve as Mucus Simulants: A Review

**DOI:** 10.3390/gels9070555

**Published:** 2023-07-07

**Authors:** Appu Vinod, Rafael Tadmor, David Katoshevski, Ephraim J. Gutmark

**Affiliations:** 1 Department of Mechanical Engineering, Ben Gurion University, Beer Sheva 84105, Israel; appu@post.bgu.ac.il; 2 Department of Civil and Environmental Engineering, Ben Gurion University, Beer Sheva 84105, Israel; davidk@bgu.ac.il; 3 Department of Aerospace Engineering & Engineering Mechanics, University of Cincinnati, Cincinnati, OH 45221, USA; gutmarej@ucmail.uc.edu

**Keywords:** mucus, synthetic hydrogels, polymers, gel networks

## Abstract

Mucus is a critical part of the human body’s immune system that traps and carries away various particulates such as anthropogenic pollutants, pollen, viruses, etc. Various synthetic hydrogels have been developed to mimic mucus, using different polymers as their backbones. Common to these simulants is a three-dimensional gel network that is physically crosslinked and is capable of loosely entrapping water within. Two of the challenges in mimicking mucus using synthetic hydrogels include the need to mimic the rheological properties of the mucus and its ability to capture particulates (its adhesion mechanism). In this paper, we review the existing mucus simulants and discuss their rheological, adhesive, and tribological properties. We show that most, but not all, simulants indeed mimic the rheological properties of the mucus; like mucus, most hydrogel mucus simulants reviewed here demonstrated a higher storage modulus than its loss modulus, and their values are in the range of that found in mucus. However, only one mimics the adhesive properties of the mucus (which are critical for the ability of mucus to capture particulates), Polyvinyl alcohol–Borax hydrogel.

## 1. Introduction

Mucus is a viscoelastic fluid produced by the epithelial secretory cells in the mucous membrane [1,2]. In the tracheobronchial tract, it serves (among other functions) to protect from foreign agents that may enter the human body as well as to hydrate these cells by coating them [3,4,5,6]. The mucus serves as a tool to capture these foreign particles, preventing their entry into the sensitive tissues and facilitating their removal through the motion of the cilia [7]. In various conditions such as chronic obstructive pulmonary disease (COPD), COVID-19, and asthma, mucus may accumulate in the lungs, and several techniques have been suggested to clear the mucus from the lungs [8,9].

Another area of interest is the adhesivity of mucus-mimicking gels that have been particularly useful for facilitating localized drug delivery [10]. The localized drug delivery is achieved either by retaining a large amount of the drug at one specific location or by providing a specific quantity of drug to one specific location on regular intervals [10]. To design such devices, there is an interest to understand the rheological and the tribological properties of the mucus. Rheological properties describe the deformation and flow behaviors of materials. Rheology deals with the responses (rheological parameters) of the material under a certain deformation stress or a flow driving force [11]. Tribological properties describe the (non-deforming) relative motion of surfaces and specifically properties such as friction, wear, lubrication, and adhesion [12].

The technical challenges encountered in developing synthetic mucus-mimicking hydrogels are mainly reproducing the rheological characteristics, the adhesive properties of the native human mucus [13], and for experiments that include biological components, also similar biocompatibility [14,15]. This review discusses different gel compositions that mimic human mucus and study their tribological and rheological properties. The criteria for selecting these hydrogels to mimic mucus include their biocompatibility, mucoadhesivity, and rheological similarity.

## 2. Native Human Mucus

Human airway mucus is composed of 97% water and 3.0% solids [16]. The solids consist of anionic glycoproteins and mucin, which give mucus its hydrogel-like nature [17].

(a) Collection of native human mucus

Obtaining native mucus in large quantities for research can be challenging [18]. One method involves using a bronchoscopy brush to collect mucus from the human airway [19,20,21,22,23]. This method has two major problems: (i) only a small volume of mucus can be collected, and (ii) it is difficult to obtain mucus from the airway without causing physical damage by the bronchoscopy brush [3]. Despite these challenges, this technique allows one for the collection of mucus from a controlled area without salivary contamination [3]. However, due to these difficulties, most rheological and tribological studies of mucus are conducted using sputum samples [3].

(b) Rheological properties of human mucus

Rheological properties play a crucial role in characterizing both the natural mucus and its synthetic versions. Characterization of the rheological properties include (i) the storage (G′) and (ii) loss moduli (G″) of mucus. The storage modulus reflects the ability of macromolecules to store energy through bending and rotation of the bonds (within and between the molecules) that constitute the mucus [24]. The loss modulus represents the amount of energy lost as the macromolecules move within the solvent [25].

The unique biophysical properties of mucus are linked to its mucin concentration, which can significantly impact its transport properties as well [26]. High molecular weight of the mucin molecules contributes to the characteristic viscoelastic properties of human mucus [26].

Theoretical analyses and experiments have been developed to study the relationship between G′ and G″, to the applied shear (ω) [27,28]. Those analyses help to determine whether the system behaves like a gel or a solution. The relation of G′ and G″ with shear frequency (ω) is given by Equation (1):

(1) $$G^{\prime }=\frac{G^{\prime \prime }}{\tan\left(\frac{n\pi }{2}\right)}=\frac{\pi }{\Gamma \left(n\right)\sin\left(\frac{n\pi }{2}\right)}S$$

Equation (1) can be rewritten as the inequality below:

G′(ω) ~ ω^n′^ and G″ (ω) ~ ω^n″^ (2)

Here, n′ and n″ are the exponents of storage and loss modulus, respectively. At the sol–gel transition point, n′ = n″ = n, as described in the references [27,29]. The exponent n is determined by the strength of the interaction between the polymer chain segments [30,31].

**Figure 1:**
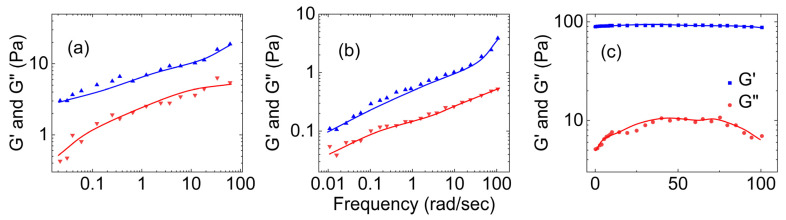
(**a**) The storage and loss moduli vs. shear applied for (**a**) the reconstituted human cervical mucus (3.5% nondialyzable solids in 0.1 M Tris-Cl-0.055 M, NaCl, pH 7.5) ((**a**) is reconstructed based on Figures 1 and 2 from Wolf et al. [24]), (**b**) the human bronchial epithelial mucus at 1% organic solids ((**b**) is reconstructed based on Figure 13 of Hill et al. [26]), and (**c**) the mucus mimetic crosslinked with glutaraldehyde (6.5 wt.%) solution ((**c**) is reconstructed based on Figure 2 of [32]).

Figure 1 shows three plots of the dependence of the storage and loss moduli on the applied shear for human native mucus of different concentrations. Figure 1a presents data for human cervical mucus acquired from Wolf et al. [24], Figure 1b presents data of human respiratory mucus acquired from Hill et al. [26], and Figure 1c presents data for an airway mucus mimetic (which has the chemical composition and concentration, bulk viscoelastic properties, and surface tension matched to that of native, non-diseased tracheal mucus) acquired from Hamed et al. [32]. All three show that their storage modulus is greater than their loss moduli. This indicates the gel-like nature of human native mucus. Also, the moduli plateau as the shear increases. This occurs because the macromolecules that compose the mucus become entangled and crosslinked, forming structures that resist deformation [24].

Hill et al. also measured the viscosity of human respiratory mucus. They found that it ranged from 0.01 Pa.s. to 60 Pa.s. The lowest viscosity was observed in mucus harvested from cell culture models, while the highest viscosity was found in mucus collected from individuals with cystic fibrosis [33].

(c) Tribological properties of human mucus

Native mucus is secreted into the airway, and it spreads over the airway epithelium, which is in contact with the cilia and the periciliary fluid layer [34]. Normally, mucus is cleared from the airway by the beating action of cilia on its surface. The viscoelasticity and the interaction of the mucus with the underlying epithelium play a crucial role in its clearance from the airway [35].

Studies by Albers et al. [35] have shown that the interfacial properties of mucus affect its transportability over the airway. The work performed to separate the mucus from the epithelium interface (work of adhesion) is calculated using the Young–Dupre equation [36], as shown in Equation (3).

(3) $$W_{SL}=\gamma_{LV}(1+\cos\theta )$$

where, $W_{SL}$ is the work needed to separate 1 cm^2^ of an interface between the mucus and the epithelium interface, θ is the contact angle that the mucus drop makes with the epithelium interface, and *γ_LV_* is the surface tension of the mucus drop.

Albers et al. reported the work of adhesion required to separate mucus from the airway duct using two techniques: du Nouy ring method [37] and Young–Dupre equation. Their findings are summarized in Table 1. 

Albers et al. discovered that an increase in the work of adhesion led to a decrease in the transportability of the mucus. 

## 3. Synthetic Mucus

Synthetic mucus has been used to better understand some of the functional properties of lung fluids. Synthetic hydrogels consist of polymeric materials to match the bulk viscoelastic behavior of mucus [32]. While not all the hydrogels discussed below are designed to mimic human airway mucus, they are widely used in biomedical applications due to their mucus-like physical properties.

### 3.1. Synthetic Mucus Prepared by Polyvinyl Alcohol

Polyvinyl alcohol (PVA) hydrogels have recently gained attention due to their hydrophilic, biodegradable, and biocompatible properties, making them a versatile material for various biomedical applications [38,39,40]. Notably, in tissue engineering, PVA hydrogels have shown promise in repairing and regenerating diverse tissues and organs, such as heart valves, corneal implants, and cartilage substitutes [41]. They also have potential as mucoadhesive and drug delivery systems [42]. Building on these applications, Singh et al. [43] investigated the mucoadhesive ability of PVA hydrogel crosslinked with sterculia gum. They discovered that the hydrogel exhibited strong adhesive strength with the mucous membrane. This suggests that the PVA hydrogel films could effectively adhere to wound sites, offering protection against pathogens that could make them candidates for drug delivery applications [43].

(a) Rheological properties of polyvinyl alcohol hydrogels

**Figure 2:**
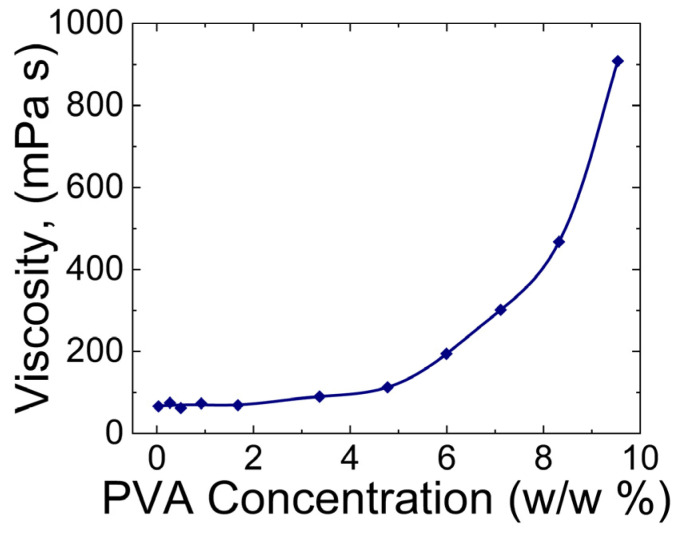
Plot of viscosity vs. concentration of the PVA hydrogel at 20 °C. (The plot is reconstructed based on Figure 1 from Krise et al. [44]).

Krise et al. [44] measured the viscosity of PVA hydrogels as a function of their concentration. As shown in Figure 2, increasing the concentrations of PVA resulted in higher viscosity of the hydrogel. This trend has also been observed in other studies [45,46,47].

**Figure 3:**
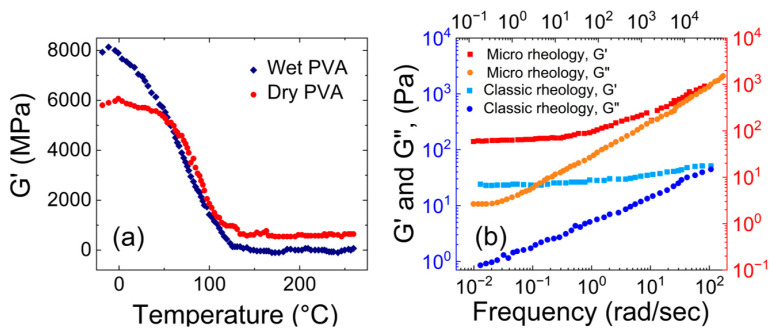
(**a**) The storage modulus vs. temperature for wet and dry PVA ((**a**) is reconstructed based on Figure 5 from Park et al. [48]), and (**b**) the storage and loss moduli vs. applied shear for a PVA hydrogel. The plots with shades of red corresponds to micro rheology, and the plots with shades of blue corresponds to macro rheology ((**b**) is reconstructed based on Figure 7 of Narita et al. [49]).

Figure 3a shows variation of the storage modulus with temperature for both dry and wet PVA hydrogels (5.0 wt.% PVA), based on studies by Park et al. [48]. Heating the samples from −20 to 260 °C resulted in a significant decrease in their storage modulus. For dry PVA, the storage modulus exhibited a steep decline until 100 °C and then remained constant. In wet PVA, the decrease in the storage modulus was gradual until 125 °C, beyond which it remained constant.

Figure 3b shows a comparison between the macro and micro rheology of chemically cross-linked PVA hydrogels (4.4 wt.% PVA), based on the study by Narita et al. [49]. They observed that at lower frequencies, the storage modulus was bigger than the loss modulus for both the macro and micro rheology. Whereas for micro rheology, at higher frequencies, the loss modulus was almost equal to the storage modulus. The macro rheology results lie within the range of the dynamic moduli observed for mucus sample studied by Wolf et al., and the results of micro rheology lie within the range of dynamic moduli results observed for mucus-mimetic samples studied by Hamed et al.

(b) Tribological properties of polyvinyl alcohol hydrogels

**Figure 4:**
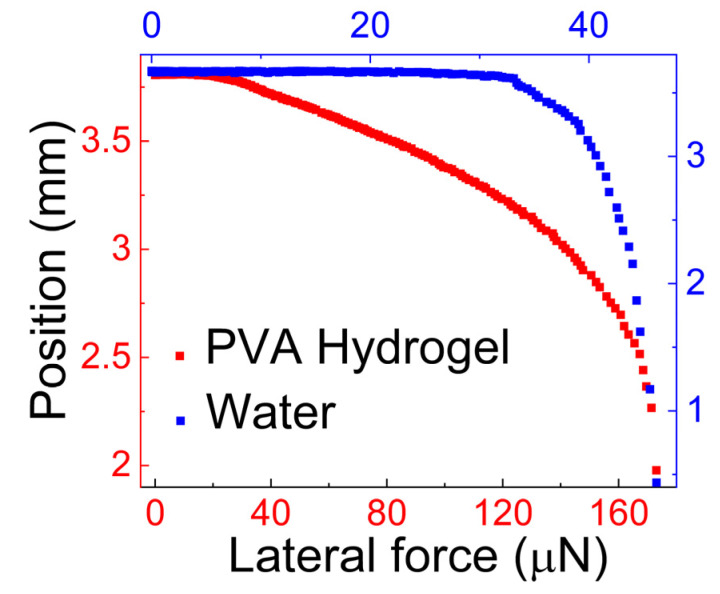
Position vs. the lateral force acting on the drop. The graph is used to determine the lateral force needed to initiate motion of the PVA hydrogel drop on the hydrophobic, octadecyl-trimethoxy-silane-coated silicon, surface.

In Figure 4, we present a new finding about the tribological properties of PVA. These results were obtained using Centrifugal Adhesion Balance (CAB) [50]. The CAB manipulates the normal and lateral forces acting on a droplet [50], which enables the measurement of the force required to slide or detach drops from solid surfaces. In Figure 4, the drop is PVA hydrogel (which represents a mucus simulant), and the solid is a hydrophobic surface, which represents a hydrophobic contaminant that may adhere to the mucus. This surface is prepared by coating octadecyl-trimethoxy-silane on silicon. In our experiment, we determined the lateral force, *f_||_*, needed to initiate the motion of a 3.0 μL PVA drop (1 wt.%) on the hydrophobic surface. Figure 4 illustrates the results of the experiment.

We compared the force required to slide the 3.0 μL hydrogel with the force required to slide a 3.0 μL water droplet. Figure 4 shows the change in the drop position as the applied centrifugal force increases. From the graph, we determined the lateral force, *f_||_*, required to slide the hydrogel drop according to the criterion mentioned earlier [51]. The lateral force, *f_||_*, for the PVA hydrogel is 111 μN, and for water, it is 33.0 μN. The higher lateral force observed for the hydrogel indicates its stronger adhesion to the hydrophobic surface compared to that of water. It highlights the good mucoadhesivity of PVA hydrogel.

### 3.2. Synthetic Mucus Prepared by Polyvinyl Alcohol Crosslinked Using Borax

The PVA–Borax hydrogel finds applications in drug delivery, wound dressing, artificial cartilage materials, and other medical uses [52]. One of the most important features of this hydrogel is, even after being broken apart, that it can reform into a single continuous piece without any additional external stimuli. This happens due to the reformation of hydrogen bonds that were cracked [53].

(a) Rheological properties of polyvinyl alcohol hydrogels crosslinked using boron

**Figure 5:**
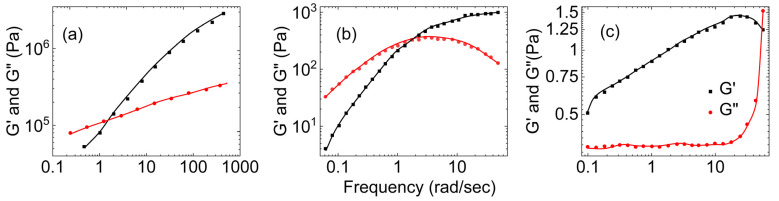
The storage and loss moduli vs. increase in the shear frequency for different compositions of PVA–Borax hydrogel: (**a**) 60 g/L of PVA with 0.28 M of borax at 60 °C ((**a**) is reproduced based on Figure 10 from Lin et al. [28]), (**b**) 4 wt.% of PVA with 0.4 wt.% of borax ((**b**) is reconstructed based on Figure 6 from Lu et al. [54]), and (**c**) 1 wt.% of PVA with 1 wt.% of borax mixed in a volumetric ratio of 10:1, respectively ((**c**) is reprinted from the supplementary material of Vinod et al. with their permission [51]).

Figure 5 shows three plots of the dependence of the storage and loss moduli on the applied shear for PVA–Borax hydrogels of different concentrations. Figure 5a presents data for 6.0 wt.% PVA solution acquired from Lin et al. [28], Figure 5b presents data for 4.0 wt.% PVA solution acquired from Lu et al. [54], and Figure 5c presents data for 1.0 wt % PVA solution acquired from Vinod et al. [51]. All three show a crossover between G′ and G″, but for Figure 5a,b, G″ is higher than G′ at lower frequencies and opposite at higher frequencies. However, in Figure 5c, G′ is bigger than G″ at the lower frequencies, and the crossover is inverted. 

The results of Lu et al. within the range of the dynamic moduli results observed for mucus-mimetic samples studied by Hamed et al., and the results of Vinod et al. lie within the range of the dynamic moduli results observed for mucus samples studied by Hill et al.

(b) Tribological properties of polyvinyl alcohol hydrogels crosslinked using boron

Cui et al. [55] conducted a study on the tribological properties of PVA–Borax hydrogel using a UMT-2 tribometer at room temperature. Their results demonstrated that pure PVA hydrogel exhibits the highest coefficient of friction, which is around 0.16. As the concentration of borax in the PVA solution was increased, the coefficient of friction decreased. The lowest value of coefficient of friction was 0.08, which was observed within the range of 0.3 to 0.4 wt.% of borax. Cui et al. also investigated the effects of normal load and sliding speed on the frictional properties of PVA–Borax hydrogel coated on stainless steel balls. They observed that as the normal load increased from 2.0 to 8.0 N, the coefficient of friction increased from a value of 0 to 0.10. This phenomenon can be attributed to the increase in the solid contact area between the hydrogel and the stainless-steel balls. As the contact area increases, a complete extrusion of water from the hydrogel occurs, which increases the coefficient of friction. Regarding sliding speed, the coefficient of friction increased with higher sliding speeds. Like the case of normal load, this too can be attributed to the compression of the hydrogel during sliding, which forces water out of the gel network. This leads to an increase in the coefficient of friction.

**Figure 6:**
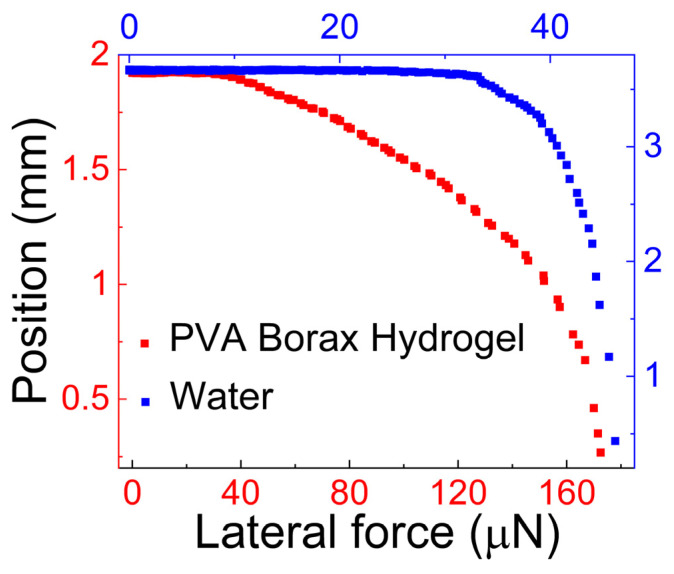
Position vs. the lateral force acting on the drop. This graph is used to determine the lateral force needed to initiate motion of the PVA–Borax hydrogel drop on the hydrophobic, octadecyl-trimethoxy-silane-coated silicon, surface.

**Figure 7:**
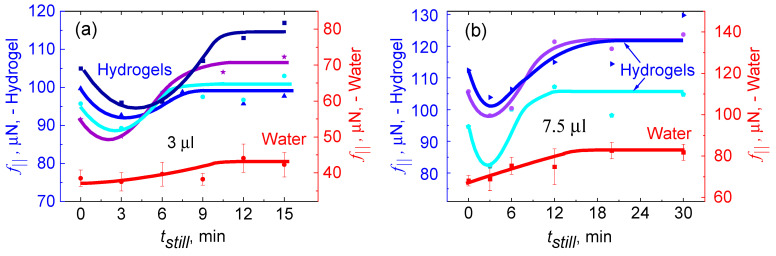
Variation of lateral force, $f_{∥}$, with the increase in still time, *t_still_*, for hydrogels (shades of blue) and for water (red color) on octadecyl-trimethoxy-silane-coated silicon surface. The dots show experimental data from different runs of (**a**) 3.0 μL and (**b**) 7.5 μL. The water data points represent an average of three different runs of the corresponding volumes. The gel data (blue lines) represent individual runs without averaging. Beyond the scatter, the different bluish curves show reproducibility of a minimum of around 3 min. The solid lines are guides to the eye. (The plots are printed after getting permission from Vinod et al. [51]).

**Figure 8:**
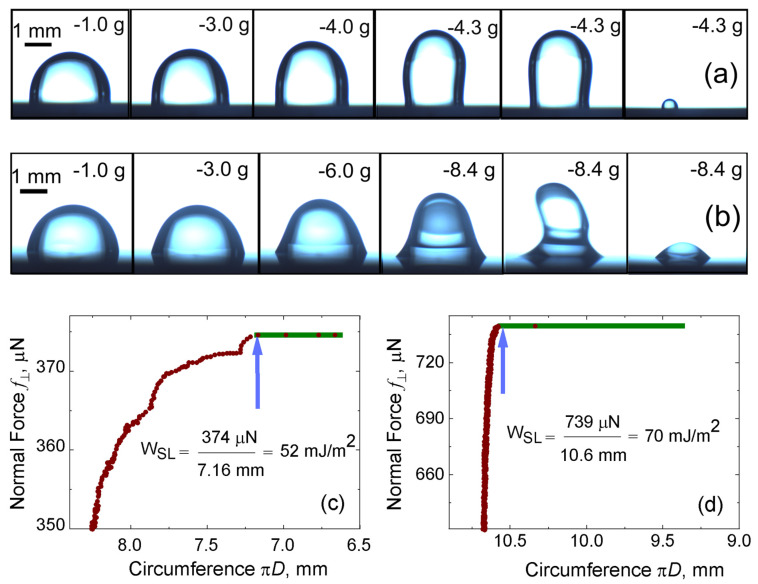
The triple line circumference of 8.0 µL drops on octadecyl−trimethoxy−silane−coated silicon surface versus the effective gravitation force pulling the drops. Image strips of drops with corresponding effective gravitation force pulling on them, (**a**) water and (**b**) PVA hydrogel (0 *t_still_*). Determination of the work of adhesion for (**c**) water drop and (**d**) hydrogel (0 *t_still_*). The blue arrows point to the data taken for the calculation of the work of adhesion, i.e., when the drops’ diameter reduces spontaneously with no increase in normal force. (The plot is printed after getting permission from Vinod et al. [51]).

Vinod et al. [51] investigated the force required to initiate motion of a 3.0 μL PVA–Borax hydrogel drop on a hydrophobic surface using CAB. The results are presented in Figure 6, Figure 7 and Figure 8. Figure 6 provides a comparison of the lateral force needed to initiate motion for the PVA–Borax hydrogel and water. The force required to initiate motion for the hydrogel is approximately 4 times higher (166 μN) than that required for water (39.0 μN).

The force versus *t_still_* plots presented as Figure 7a shows that the lateral retention force, $f_{∥}$, necessary to initiate sliding of the hydrogel drops consistently exceeds that of pure water. This observation aligns with the adhesive properties of mucus, which tends to capture hydrophobic particles. Notably, the hydrogel exhibits such strong adhesion that it does not fully detaches from the solid silanized silicon surface on which it slides. Please refer reference [51] to read about Figure 7 in detail.

Figure 8a,b illustrates how the water and the hydrogel drops respond to an increase in the normal force acting on it, respectively. The data from Figure 8c,d indicate that a higher work of adhesion is needed to separate the hydrogel from the hydrophobic surface in comparison to that of water. 

The work of adhesion calculated for water is 52 mJ/m^2^ (as shown in Figure 8c), and the work of adhesion calculated for PVA–Borax hydrogel is 70 mJ/m^2^ (as shown in Figure 8d). 

### 3.3. Synthetic Mucus Prepared by Using Guar Gum

Guar gum exhibits high shear viscosities, and its hydrogel solutions display shear-thinning behavior [56]. Guar gum finds practical applications in the food industry, oil recovery, and in skin care industries [57]. It serves as a mucus-like agent for colon delivery and as a matrix for oral solid dosage forms [58,59]. Due to its branched structure, guar gum readily hydrates, but it does not allow extensive hydrogen bonding between the guar macromolecules [60].

**(i)**  **Guar gum with scleroglucan**

Scleroglucan is a water-soluble polysaccharide that finds various commercial applications in secondary oil recovery, ceramic glazes, food industry, and cosmetics [58].

(a) Rheological properties of guar gum and scleroglucan mucus simulant

Zahm et al. [61] studied the viscoelastic properties of a hydrogel composed of guar gum and scleroglucan (0.5 wt.% of galactomannan). They observed a significant decrease in the hydrogel’s viscosity as the shear rate increased. The decrease in viscosity is attributed to changes in the physical crosslinking of the macromolecules that constitute the hydrogel. This molecular bond breakage and associated viscosity reduction have also been reported by Quedama and Droz [62].

**Figure 9:**
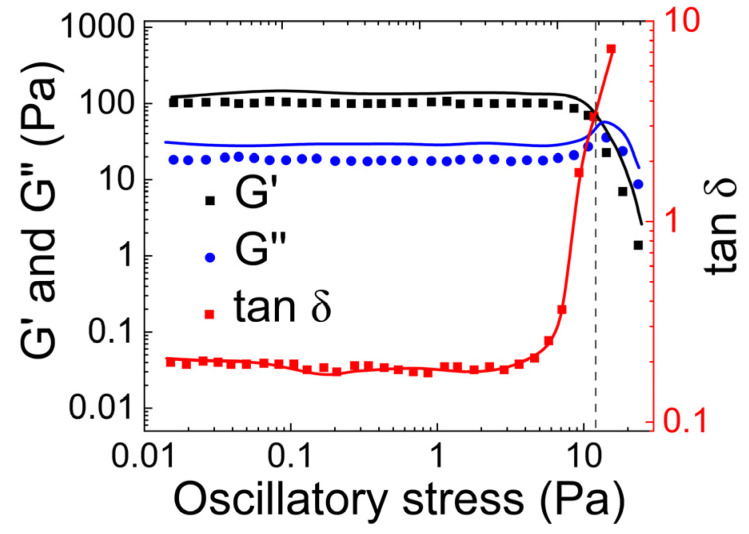
The storage (G′) modulus, loss modulus (G″), and the loss factor for guar gum scleroglucan hydrogel (1.5 wt.% concentration of scleroglucan) as a function of the oscillatory stress applied. (The plot is reconstructed based on Figure 4 from Lafforgue et al. [63]).

Figure 9 shows the variation of the storage (G′) and loss (G″) moduli of the mucus as a function of the oscillating stress amplitude based on a study by Lafforgue et al. [63]. They synthesized the same hydrogel as Zahm et al. Their results indicate that the G′ values of the mucus simulant are consistently higher than its G″ values, until the yield point. Beyond the yield point, the behavior of G′ and G″ changes as the hydrogel network starts to breakdown. At a certain point, G″ surpasses G′, indicating that the material becomes more viscous than elastic.

Lafforgue et al.’s results are similar to Zahm et al. and Mezger et al. [64] regarding an overshoot in the viscous modulus after the yield point. This overshoot occurs due to the energy dissipation resulting from the breakage of the network that holds the hydrogel together. The variation in G′ and G″ with oscillatory stress and the overshoot of G″ at the yield point are consistent with the findings of Lai et al. [65].

(b) Tribological properties of mucus simulated using guar gum and scleroglucan mucus simulant

Lafforgue et al. conducted measurements of the surface tension of the mucus simulant, consisting of guar gum and scleroglucan, using a du Nouy ring [63]. Their findings indicated that the surface tension of the simulated mucus varied from 72 to 90 mN/m, corresponding to scleroglucan concentrations ranging from 0.5% to 2.0%, respectively.

**(ii)**  **Guar gum with borax**

Researchers [66,67] investigated how the rheological properties of the guar gum and borax hydrogel depend on various factors, such as polymer compositions, temperature, and pH conditions. Borax plays a crucial role in promoting rapid gelation of guar gum by forming crosslinks (with a lifetime in the order of seconds), which contributes to the self-healing properties of this network. The reversible chemical bridges formed between the chains of guar gum through crosslinking with borax are responsible for its unique characteristics [57].

(a) Rheological properties of guar gum with borax

**Figure 10:**
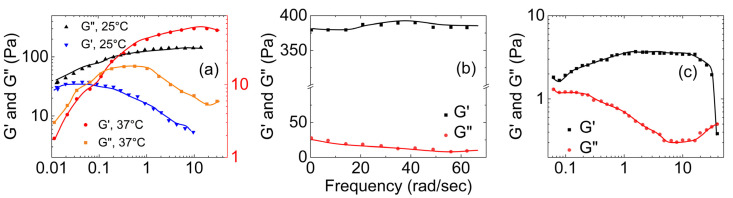
(**a**) The storage modulus (G′) and loss modulus (G″) of guar gum with borax vs. shear frequency at 25 °C and 37 °C. The black shades represent 25 °C, and the red shades represent 37 °C ((**a**) is reproduced based on Figures 4 and 5 of Coviello et al. [57]), and (**b**,**c**) the storage modulus G′ and loss modulus G″ of guar gum with borax hydrogel versus shear frequency ((**b**) is reconstructed using Figure 6 from Pan et al. [68]), and (**c**) is reconstructed based Figure 7 of Sun et al. [69].

Figure 10 shows three plots of the dependence of the storage and loss moduli on the applied shear for guar gum with borax hydrogels of different concentrations. As shown in Figure 10a, Coviello et al. [57] characterized the rheological properties of the guar gum–borax hydrogel at 25 °C and 37 °C. At 25 °C, the storage modulus (G′) was consistently higher than the loss modulus (G″). This indicates the elastic nature of the hydrogel. However, as the temperature increased to 37 °C, the loss modulus was higher than the storage modulus at lower frequencies. As the frequency increased (at a crossover point of ω = 0.2 rad/s), the storage modulus surpassed the loss modulus.

**Figure 11:**
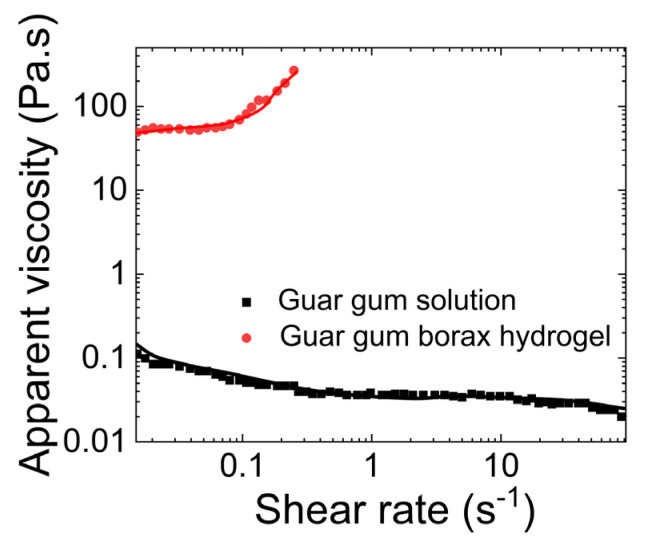
Comparison between the apparent viscosities and shear rate of guar gum solution to guar gum−borax hydrogel. (The plot is reproduced based on Figure 3 of Sun et al. [69]).

Figure 10b shows the dependence of the rheological moduli on shear applied for gum–borax hydrogel (15 g/L guar gum solution) based on a study by Pan et al. [68]. They observed that the storage modulus of the hydrogel was always bigger than the loss modulus. Moreover, G′ remained relatively constant with an increase in frequency, while G″ exhibited a slight decrease. Figure 10c shows the dependence of rheological properties of the guar gum–borax hydrogel (2.5 g/L guar gum solution) on shear applied, based on a study by Sun et al. [69]. The storage modulus (G′) of the hydrogel was higher than the loss modulus (G″). The crossover frequency occurred at 36 rad/s. They also studied the dependence of its apparent viscosity on the shear rate. As shown in Figure 11, they found that the viscosity of the guar gum solution remained almost at 0.1 Pa.s, even with the increasing shear applied. In contrast, the apparent viscosity of the guar gum with borax increased with increasing shear rate.

(b) Tribological properties of mucus simulated using guar gum and borax

Pan et al. [68] studied the adhesive properties of a borax crosslinked guar gum hydrogel. Adhesion tests were performed using a digital tensile machine. In their study, the hydrogel was applied onto a metal sheet coated with a surface-mimicking human skin. Then, the hydrogel was pulled until it detached from the ‘human skin surface’. The results indicated that a work of adhesion of 2.5 KPa was required to detach the hydrogel from the ‘human skin mimicking surface’. This observation suggests that the hydroxyl groups in the crosslinked guar gum hydrogel have the ability to form hydrogen bonds with the ‘human skin surface’.

### 3.4. Synthetic Mucus Based on Polyglycerol

Linear polyglycerol is used as a base for developing synthetic hydrogels due to the presence of pendant hydroxyl groups. Bej et al. [13] and other researchers [70,71] reported the effectiveness of sulfated linear polyglycerol as an inhibitor against respiratory diseases such as HSV-1 and COVID-19.

(a) Rheological properties of linear polyglycerol based synthetic hydrogel

Sharma et al. [72] investigated the viscoelastic properties of three different Mucus Inspired Hydrogels (MIHs), named MIH-1, MIH-2, and MIH-3. These hydrogels contain reversible, redox-responsive bonds similar to those found in native mucus.

MIH-1, MIH-2, and MIH-3 differ in the linear polymers (polyglycol) used as their backbones and also on the crosslinker to polymer ratio used to synthesize them. Table 2 lists all the MIHs with their corresponding polymer backbone and also with their crosslinker to polymer ratio.

**Figure 12:**
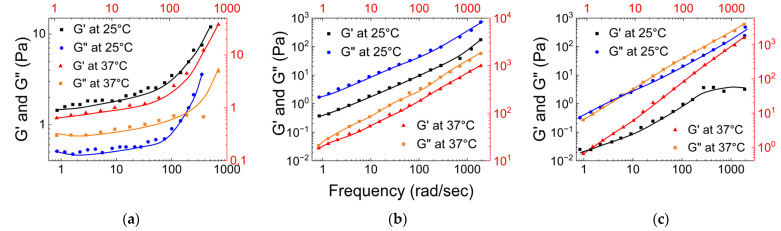
(**a**) The storage modulus and loss moduli of MIHs hydrogels vs. shear applied at 25 °C and 37 °C. The black shades represent experiments at 25 °C, and the red shades represent experiments at 37 °C (**a**) for MIH-1, (**b**) for MIH-2, and (**c**) for MIH-3 ((**a**) is reconstructed based on Figure 4 of Sharma et al. [72], (**b**) is reconstructed based on Figure 2 of Sharma et al. [72], and (**c**) is reconstructed based on Figure 2 of Sharma et al. [72]).

As shown in Figure 12a, for MIH-1 (data presented in Figure 12a is an average of MIH-1a, MIH-1b, MIH-1c, and MIH-1d), the storage modulus was higher than the loss modulus, and both G′ and G″ increased by more than a factor of 10 as the temperature increased, with a more pronounced effect on G′. This indicates that MIH-1 is a hydrogel that is dominated by its elastic nature, and as the temperature increases, the elastic property of the gel increases. Also, the dynamic moduli results of MIH-1 lies within the range of the dynamic moduli results observed for native mucus samples studied by Wolf et al.

For MIH-2, as shown in Figure 12b, both G′ and G″ exhibited a linear increase with shear, and the loss modulus was always higher than its storage modulus. Both these trends remained the same even with an increase in the temperature. In Figure 12c, it is evident that the storage and loss moduli of MIH-3 increase almost linearly with shear rate. Same as MIH-2, the loss modulus for MIH-3 hydrogel surpasses its storage modulus. This indicates that the hydrogels, MIH-2 and MIH-3, are dominated by their viscous characteristics.

**Figure 13:**
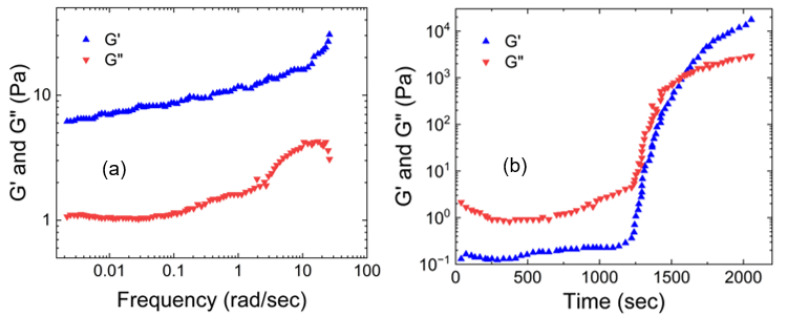
(**a**) The storage modulus (G′) and loss modulus (G″) of 3.6 wt.% dPGS hydrogels as a function of shear applied ((**a**) is reconstructed using Figure 4 of Lospichl et al. [74]), and (**b**) the evolution of the storage (G′) and loss (G″) moduli during the polymerization and hardening of 0.1 wt.% of glycerol glycidyl ether ((**b**) is reconstructed based on Figure 4 of Ekinci et al. [75]).

Lospichl et al. [74] examined the viscoelastic properties of polyglycerol sulfate hydrogels. As shown in Figure 13a, they observed pronounced gel-like behavior in all the tested gel samples, with G′ significantly exceeding G″. Also, the dynamic moduli results of polyglycerol sulfate hydrogels lie within the range of the dynamic moduli results observed for native mucus samples studied by Wolf et al.

Ekinci et al. [75] investigated the rheological properties of a polyglycerol-based polymer network using a rheometer equipped with an external UV-light source. As demonstrated in Figure 13b, they observed that G″ surpassed G′, indicating a viscous fluid behavior.

The key distinction among the systems studied by the three groups (Sharma et al., Lospichl et al., and Ekinci et al.) is that Sharma et al. and Lospichl et al. employed linear polyglycerol-based hydrogels, while Ekinci et al. used branched polyglycerol-based hydrogels.

(b) Tribological properties of linear glycerol-based polymer

Orafai et al. [76] investigated the surface energy of poly (glycerol adipate) polymers by measuring their contact angles with different test liquids [77,78]. Later, they plugged these values into the Fowkes equation [79] to calculate the surface energy of the polymer surface. Table 3 shows the surface energy of the polymer measured with a change in the adipate concentration in the polymer.

From Table 3, we can see that the lowest surface energy for the surface was noticed when the adipate concentration was 40%.

### 3.5. Synthetic Mucus Prepared by Polyacrylic Acid Hydrogels/Carbopols

Polyacrylic acid (PAA) hydrogels attracted considerable attention in the recent years due to their unique properties (ability to form gels and pH sensitivity) [80], and it has found potential applications in drug delivery, tissue engineering, biosensors, and other biomedical applications [81]. PAA hydrogels are able to absorb large amounts of water while holding their structural integrity [82].

Researchers have developed a polymeric combination called Carbopols using PAA [83]. They are prepared by crosslinking polyacrylic acid polymers with different crosslinkers [84]. Rheological analysis of Carbopol is important as its mucoadhesive ability significantly depends on the rheological properties of the hydrogel [85,86,87]. Early studies on Carbopols showed that their distinct rheological properties depend on the entanglements of the high molecular weight polyacrylate molecules.

(a) Rheological properties of polyacrylic acid hydrogels

**Figure 14:**
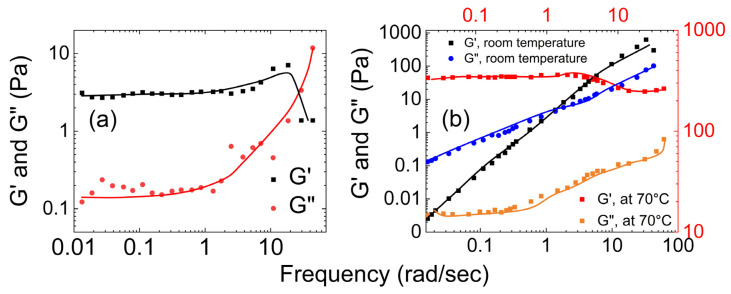
(**a**) The storage modulus (G′) and loss modulus (G″) vs. shear applied for a sample containing 4.0 wt.% of Carbopol ((**a**) is reconstructed based on Figure 4 from Kim et al. [88]), and (**b**) the frequency sweep of Carbopol gels (4 wt.%) in water prepared at room temperature and at 70 °C. The black shades represent hydrogel samples synthesized at room temperature, and the red shades represent hydrogel samples synthesized at 70 °C ((**b**) is reconstructed based on Figures 3 and 7 from Bonacucina et al. [89]).

Kim et al. [88] conducted a study on the rheology of Carbopol (4.0 wt.% PAA). They measured the storage (G′) and loss (G″) moduli in the linear viscoelastic regime during frequency sweep tests. Figure 14a shows that the storage modulus G′ consistently exceeded its loss modulus G″ for Carbopol. This low storage modulus was attributed to the flexible network structure and high-water content of the Carbopol macromolecule. At low frequencies, PAA hydrogels exhibited predominantly viscous behavior (higher loss modulus), while at high frequencies, the elastic nature dominated (higher storage modulus). Carbopol samples studied by Kim et al. exhibited dynamic moduli results that lie within the range of the dynamic moduli results of native mucus samples studied by Wolf et al.

Bonacucina et al. [89], investigated the rheological properties of Carbopol as a function of shear and temperature. They compared the physical properties of two Carbopol samples, one synthesized at room temperature and the other at 70 °C. Figure 14b illustrates that the sample synthesized at 70 °C displayed gel-like behavior, with the storage modulus (G′) consistently surpassing the loss modulus (G″) across the entire frequency range tested. Both the moduli remained independent with the increase in the frequency.

This frequency sweep test indicated that heating transforms Carbopol from a low viscosity semi-dilute solution to a gel-like structure, likely due to increased polymer–solvent interactions. Conversely, the frequency sweep test conducted on samples prepared at room temperature revealed that the loss modulus consistently exceeded the storage modulus, which is typical for a semi-dilute polymer solution. Therefore, the Carbopol synthesized at room temperature by Bonacucina et al. seems not to exhibit gel-like behavior.

**Figure 15:**
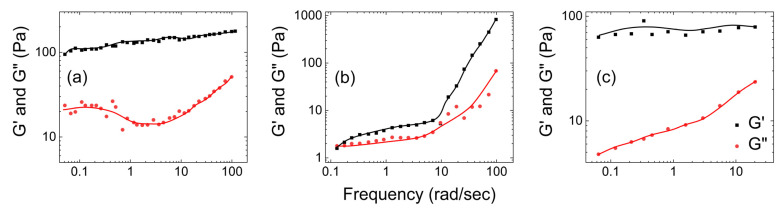
(**a**) The storage modulus (G′) and loss modulus (G″) as function of shear applied for pure Carbopol at concentrations in the viscoelastic linear regime of (**a**) 0.25 wt.%, (**b**) 0.04 wt.%, and (**c**) 0.2 wt.% ((**a**) is reconstructed based on Figure 8 of Baek et al. [90], (**b**) is reconstructed based on Figure 1 of Schenck et al. [91]), and (**c**) is reconstructed based on Figure 3 of Vicente et al. [92].

Figure 15 shows three plots of the dependence of the storage and loss moduli on the applied shear for Carbopol hydrogels of different concentrations. Figure 15a shows that the storage modulus (G′) was always bigger than the loss modulus (G″) for Carbopol at concentration of 0.25 wt.%., based on results by Baek et al. [90]. This indicates the elastic behavior of Carbopol.

Figure 15b shows the rheological properties of Carbopol hydrogels with concentrations ranging from 0.025% to 0.05% based on studies by Schenck et al. [91]. They observed that both the storage and the loss moduli were dependent on the frequency of the applied shear. For all gel concentrations, both the moduli increased as the shear frequency increased from 0.5 to 105 rad/s. A significant increase in G′ and G″ was noticed at 10 rad/s, and continued up to frequencies of 105 rad/s. The sharp increase in the rheological moduli values at 10 rad/s was attributed to the breakdown of the gel structure, which indicates the gel’s ability to relax at high deformation frequencies. This behavior is commonly observed in semiflexible polymer networks [93] and mucus simulants [61]. Figure 15c shows the rheological studies on Carbopol at 35 °C, based on studies by Vicente et al. [92]. They used a concentration of 0.1% of Carbopol in the hydrogel. The storage values were consistently higher than the loss values. The storage modulus value remained nearly constant throughout the frequency range, with a slight increase in the loss modulus values around 10 rad/s. This observation aligns with the findings of Schenck et al., who also reported a sharp increase in the loss modulus above 10 rad/s.

(b) Tribological properties of polyacrylic acid hydrogels

The surface tension of Carbopol hydrogel was expected to be close to that of water (~72 mN/m) due to its high-water content. Furthermore, Carbopol is not surface active, which further supports this expectation. Schenck et al. [91] conducted experiments to measure the surface tension of the hydrogel at various concentrations of Carbopol. Table 4 presents the surface tensions observed for different concentrations of Carbopol.

**Figure 16:**
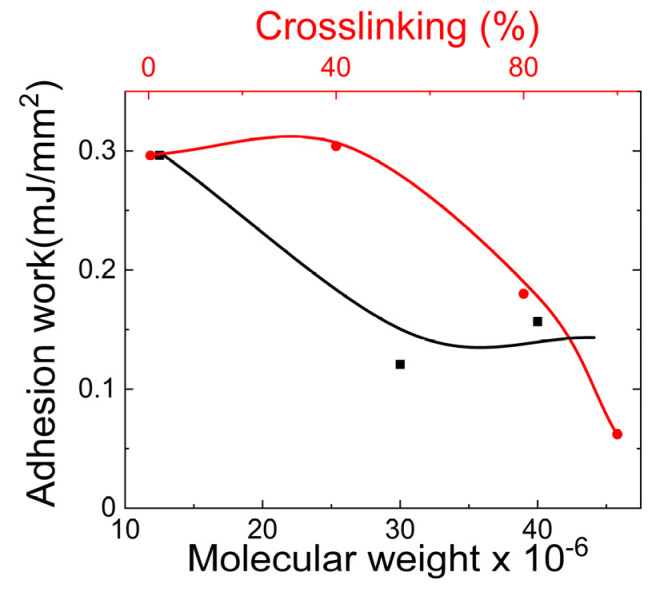
The adhesion work required for separating Carbopol hydrogels from a tanned leather substrate. The black shade represents the variation in the adhesion work with increasing molecular weight of the Carbopol, while the red shade demonstrates the changes in the adhesion work with increased crosslinking between polymer molecules.

Fuente et al. [94] studied the work of adhesion needed to detach Carbopol hydrogel from tanned leather substrates. The work of adhesion was determined using a tensile tester (Lloyd, Instruments LR 5K). As shown in Figure 16, they found that an increase in the Carbopol concentration in the hydrogel led to a decrease in the work of adhesion. Also, they found that an increase in the crosslinking between the polymer molecules of the hydrogel led to a decrease in the work of adhesion.

Chau et al. [95] studied the dependence of pH on the coefficient of friction of polyacrylic acid hydrogel. They conducted tribological experiments using a linear reciprocating tribometer and found that the coefficient of friction of the hydrogels could be altered by varying the hydrogel’s pH and acrylic acid concentration. The friction coefficients ranged from 0.17 ± 0.01 (at pH = 0.35) to 0.005 ± 0.001 (at pH = 7) and decreased with increasing pH across all acrylic acid concentrations.

Please see the Supplementary Material for a detailed description of the methodol-ogies employed by various groups to synthesize different mucus mimicking hydrogels (Table S1), details about the equipment utilized by various research groups for study-ing the rheological moduli of mucus mimicking hydrogels (Table S2), and details about the equipment utilized by various research groups for studying the tribological proper-ties of mucus mimicking hydrogels (Table S3).

## 4. Discussion and Summary

In drug delivery systems, hydrogels are used as carriers to encapsulate and release therapeutic agents [96,97]. The rheological moduli of the hydrogel influence the drug release kinetics, and the release behavior of the loaded drug [98]. A hydrogel with a higher storage modulus (G′) is generally associated with a more rigid structure, leading to a controlled release rate of the drug [99,100]. Along with this, the rheological moduli of hydrogels also affect their interactions with biological tissues [101]. The mechanical properties of natural tissues are better imitated by soft and flexible hydrogels with lower storage modulus [102]. Such hydrogels are selected for tissue engineering applications, where the hydrogel’s scaffold provides a suitable environment for cell growth and tissue regeneration [102].

The rheological moduli of a hydrogel significantly affect its mucoadhesive properties [103]. In the context of mucoadhesion, a hydrogel with higher storage modulus promotes better contact and adherence to the mucosal surface, since it has necessary strength and cohesion to prevent detachment and withstand shear forces [104].

Among the various mucus-mimicking hydrogels reviewed, only one demonstrated the particle capturing ability comparable to that of native mucus. The challenges in developing such mucus simulants can be attributed to several factors, such as the complex and dynamic nature of natural mucus, the difficulty in replicating its intricate microstructure, and the intricate biological interactions involved [105]. The composition of native mucus (the ratio by which mucins, lipids, enzymes, salts, and water are mixed) also plays a crucial role in particle capture [7]. It could be difficult to accurately recreate the precise composition of native mucus in synthetic hydrogels. Furthermore, accurately mimicking the mucus’s viscoelastic properties and its interactions with epithelial cells and particles adds further complexity to the development of mucus simulants [106].

Table 5 summarizes the rheological properties of the hydrogels that we reviewed.

Table 6 presents a comparative analysis between the rheological moduli of human native mucus and various mucus-mimicking gels. The study by Wolf et al. focuses on reconstituted human cervical mucus, while Hamed et al.’s research involves a mucus-mimetic hydrogel synthesized using components resembling tracheal mucus.

Table 7 gives a summary of the tribological properties of the hydrogels that we reviewed.

Mucus-mimicking hydrogels also have applications in various fields other than tissue engineering and drug delivery. One such area is environmental remediation [107]. These hydrogels could be used to selectively bind and remove pollutants from water sources [108,109]. By imitating the adhesive and filtration properties of mucus, these hydrogels could be used as effective filters for purifying contaminated water and contributing to cleaner ecosystems [110].

Additionally, mucus-mimicking hydrogels may be used in the development of biosensors and next-generation sensors [111]. By mimicking the mucus barrier present in various tissues, these hydrogels can aid in the creation of highly sensitive and selective sensors for detecting specific molecules or pathogens. This could have implications in medical diagnostics [112], food safety [113], and environmental monitoring [114]. Furthermore, mucus-mimicking hydrogels have the potential to advance the field of soft robotic skin-like stretchable sensors and prosthetics [115].

## 5. Conclusions

Synthetic hydrogels designed to replicate the physical properties of mucus found in the human body are reviewed. These hydrogels typically consist of water-swollen polymers and other substances that bind the polymer chains together. One of their key characteristics is the ability to accurately mimic the rheological and the tribological behavior of natural human mucus with a higher storage modulus than loss modulus. This unique capability has led to a wide range of applications for these hydrogels, including their use as drug delivery systems and for mucoadhesive purposes. The review highlighted the features that make them useful in medical research, particularly in the study of respiratory diseases like cystic fibrosis, asthma, and chronic obstructive pulmonary disease. Along with biomedical applications, mucus-imitating hydrogels also find applications in the field of soft robotics stretchable sensors and environmental engineering.

## Figures and Tables

**Table 1:** Work of adhesion calculated using two different methods for mucus samples collected from two different sources.

Mucus Samples Collected from People with:	Calculated Work of Adhesion to Separate Mucus from Airway Duct	
	du Nouy Ring	Using Equation (3)
Cystic fibrosis	140 ± 30 mN/m	160 ± 20 mN/m
Chronic bronchitis	130 ± 20 mN/m	150 ± 20 mN/m

**Table 2:** The table shows the polymers used and the ratio between the crosslinker to polymer ratio used to synthesize different mucus inspired hydrogels. LPG_5_(SH)_2_ is obtained after acidic and basic hydrolysis of ethoxy ethyl and thiourea group, PEG_3_(SH)_2_ is synthesized following an approach modified from the original report from Mahadevagowda and Stuparu [73]. A detailed description of the preparation of PEG_6_(SH)_2_ is given in reference [72]. The rheological properties of these MIHs were characterized by storage (G′) and loss (G″) moduli obtained from oscillatory shear experiments conducted at 25 °C and 37 °C.

MIH Studied	Polymer Used as Backbone	Crosslinker to Polymer Ratio
MIH-1	LPG_5_(SH)_2_	MIH-1 shown in the plot (Figure 12a) is an average of the ratios 1:3, 1:7, 1:10, 1:14.
MIH-2	PEG_3_(SH)_2_	1:3
MIH-3	PEG_6_(SH)_2_	1:3

**Table 3:** The table shows the change in surface energy of the polymer as the adipate concentration in it changes.

Adipate Concentration (%)	Surface Energy (mJ/m^2^)
0	62.4
20	60.7
40	31.4
100	57.3

**Table 4:** Surface tension of the hydrogels at different concentrations of Carbopol.

Carbopol Concentration (wt.%)	Surface Tension (mN/m)
0.025	72.3 ± 0.10
0.04	71.7 ± 0.20
0.05	71.8 ± 0.40

**Table 5:** Details of the rheological properties of the hydrogels considered in this review.

Hydrogel Used	Research Conducted by	Comments by the Research Group on the Hydrogel’s Rheological Properties
Native mucus	Wolf et al. [24]	G′ > G″
	Hill et al. [26]	G′ > G″
Polyvinyl alcohol	Park et al. [48]	G′ decreased as the temperature increased.
	Narita et al. [49]	At lower frequencies, G′ < G″, and at higher frequencies, G′ > G″.
	Lu et al. [54]	At lower frequencies, G″ > G′, and at higher frequencies, G′ > G″
Polyvinyl alcohol with boron	Lin et al. [54]	At lower frequency G′ < G″, and at higher frequency the G′ > G″.
	Vinod et al. [51]	G′ > G″
Guar gum borax	Pan et al. [68]	G′ > G″
	Sun et al. [69]	G′ > G″
	Sharma et al. [72]	For MIH-1, G′ > G″ For MIH-2 and MIH-3, G″ > G′
Polyglycerol	Lospichl et al. [74]	G′ > G″
	Ekinci et al. [75]	G′ < G″
	Kim et al. [88]	G′ > G″
Polyacrylic acid	Bonacucina et al. [89]	G′ > G″
	Baek et al. [90]	G′ > G″
	Schenck et al. [91]	G′ > G″
	Vicente et al. [92]	G′ > G″

**Table 6:** Comparison of rheological moduli for human native mucus and mucus-mimicking hydrogels.

Rheological Moduli of Native Airway Mucus		Group	Hydrogels with Rheological Moduli Falling in the Range of Native Airway Mucus
Storage Modulus (G′, Pa)	Loss Modulus (G″, Pa)		
~1	~0.1	Hill et al. [26]	PVA Borax (1.0 wt.%)
~10	~1.0	Wolf et al. [24]	2. PVA (4.4 wt.%) 3. MIH-1 4. Polyglycerol sulfate 5. Carbopol (4.0 wt.%)
~100	~10	Hamed et al. [32]	PVA (4.4 wt.%) PVA (4.0 wt.%) Guar gum (0.5 wt.%) with scleroglucan (1.5 wt.%) Guar gum with borax (0.1 M) Carbopol (0.25 wt.%, 0.04 wt.%, 0.2 wt.%).

**Table 7:** Details of the tribological properties of the hydrogels considered in this review.

Hydrogel Used	Research Conducted by	Comments by the Research Group on the Hydrogel’s Tribological Properties
Native mucus	Albers et al. [35]	Measured the work of adhesion needed to move native mucus from the airway duct: Using the ring method, the values ranged from 130 ± 20 mN/m to 140 ± 30 mN/m. Using the contact angle method, 150 ± 20 mN/m-160 ± 20 mN/m
	King et al. [61]	As the work of adhesion increased, the transportability of the mucus decreased.
Polyvinyl alcohol	Vinod et al. [51]	The lateral force, *f_||_*, for sliding = 111 μN.
Polyvinyl alcohol with boron	Cui et al. [55]	The coefficient of friction (COF): PVA hydrogel, COF = 0.16. PVA–Borax hydrogel, COF = 0.08.
	Vinod et al. [51]	The lateral force, *f_||_*, needed to slide the hydrogel drop was around 166 μN.
Guar gum with scleroglucan	Lafforgue et al. [63]	The surface tension of the hydrogel = 72 to 90 mN/m.
Guar gum borax	Pan et al. [68]	The work of adhesion required to detach the hydrogel from the ‘human skin-mimicking surface’ was 2.5 KPa.
Polyglycerol	Orafai et al. [76]	Surface energy of the hydrogel = 31.4 mJ/m to 62.4 mJ/m.
Polyacrylic acid	Schenck et al. [91]	Surface tensions were recorded for different weight percentage of polymer in the hydrogel: 0.025%—72.30 ± 0.10 mN/m 0.04%—71.70 ± 0.20 mN/m 0.05%—71.80 ± 0.40 mN/m
	Chau et al. [95]	The coefficient of friction (COF) was recorded based on the pH of the hydrogel: pH = 0.35, COF = 0.17 ± 0.01 pH = 7.00, COF= 0.005 ± 0.001

## Supplementary Materials

The following supporting information can be downloaded at: https://www.mdpi.com/article/10.3390/gels9070555/s1. Three tables are presented in the supporting material: Table S1 that presents the methodologies employed by various groups to synthesize different mucus-mimicking hydrogels, Table S2 that displays the different equipment utilized by various research groups for studying the rheological moduli of mucus-mimicking hydrogels, and Table S3 displays the different equipment utilized by various research groups for studying the tribological properties of mucus-mimicking hydrogels. References [116,117] are cited in the Supplementary Materials.
