# Supplementary material for: Gels That Serve as Mucus Simulants: A Review
Source: Gels. 2023 Jul 7;9(7):555. doi: 10.3390/gels9070555 (PMC10379079; doi:10.3390/gels9070555)
Supplement: Supplementary file 1 [file gels-09-00555-s001.zip › gels-2455077-supplementary.pdf]

Supporting information for:
Gels that Serve as Mucus Simulants: A review

Appu Vinod [1], Rafael Tadmor [1], David Katoshevski [2] and Ephraim. J. Gutmark [3]

1. Ben Gurion University, Department of Mechanical Engineering, Beer Sheva, Israel
2. Ben Gurion University, Department of Civil and Environmental Engineering, Beer Sheva, Israel
3. Aerospace Engineering & Engineering Mechanics, University of Cincinnati, Cincinnati, OH

*Corresponding author email: tadmorr@bgu.ac.il

Abstract

Mucus is a critical part of the human body's immune system which traps and carries away various particulates such as anthropogenic pollutants, pollen, viruses etc. Various synthetic hydrogels have been developed to mimic mucus, using different polymers as their backbones. Common to these simulants is a three-dimensional gel network which is physically crosslinked and is capable of loosely entrapping water within. Two of the challenges in mimicking mucus using synthetic hydrogels include the need to mimic the rheological properties of the mucus and its ability to capture particulates (its adhesion mechanism). In this paper, we review the existing mucus simulants and discuss their rheological, adhesive and tribological properties. We show that most, but not all, simulants indeed mimic the rheological properties of the mucus: like mucus, most hydrogel mucus simulants reviewed here demonstrated a higher storage modulus than its loss modulus, and their values are in the range of that found in mucus. However, only one mimics the adhesive properties of the mucus (which are critical for the ability of mucus to capture particulates), Poly vinyl alcohol - Borax hydrogel.

Keywords: Mucus, synthetic hydrogels, polymers, gel networks.

Table S1 presents the methodologies employed by various groups to synthesize different mucus-mimicking hydrogels.

Table S1. Methods for preparing hydrogels reviewed in this study.

Hydrogel	Group	Hydrogel's preparation
Human airway mucus	Wolf et al. [1]	Cervical mucus was directly collected into a 1 ml disposable syringe.
	Hill et al. [2]	Mucus was harvested from primary human bronchial epithelial cell cultures.
Poly vinyl alcohol	Krise et al. [3]	Aqueous PVA solutions of concentrations 0-10 wt. % was prepared.
	Park et al. [4]	An aqueous PVA solution of 5 wt. % was crosslinked using glutaraldehyde.
	Narita et al. [5]	An aqueous PVA solution of 4.4 wt % was crosslinked with glutaraldehyde at acidic condition.
	Vinod et al.[6]	An aqueous PVA solution of 1.0 wt. % was frozen and thawed to prepare the hydrogel.
PVA Borax	Lu et al.[7]	An aqueous PVA solution of 4.0 wt% was crosslinked with 0.4 wt.% of borax solution.
	Lin et al. [8]	An aqueous PVA solution (60 g/L) was crosslinked with 0.28 M of borax solution.
	Cui et al. [9]	Hydrogels were prepared by adding different concentrations of borax to a 15 wt % PVA solution.
	Vinod et al. [6]	1 wt. % of PVA solution was mixed with 1 wt.% of borax solution in a 10:1 volumetric ratio.
Polyglycerol	Sharma et al. [10]	Different mucus inspired hydrogels were synthesized using homobifunctionalized linear polyglycerol chains with thiol groups at both ends.
	Lospichl et al. [11]	Hydrogels were synthesized using homobifuntional polyethylene glycol-cyclooctyne and dendritic polyglycerol sulfate azide.
	Ekinci et al. [12]	Hydrogels were synthesized by photopolymerization of Glycerol glycidyl ether monomer.
	Le et al. [13]	An aqueous glycerol solution was prepared by adding 10-50 wt % of deionized water into glycerol liquid.
	Orafai et al. [14]	Poly (glycerol-adipate) (PGA) polymers and a range of substituted ones were synthesized. The substituted ones were prepared with either C_8 acyl group or Tryptophan.
Polyacrylic acid	Kim et al. [15]	A 4 wt. % poly acrylic acid polymer solution was prepared.
	Bonacucina et al.[16]	A certain amount of Carbopol was dispersed in water, polyethylene glycol and glycerine respectively.
	Schenck et al. [17]	Carbopol hydrogels were neutralized to pH 7.4 with a 2 N NaOH solution serving as the neutralizing agent.
	Baek et al.[15]	A master solution of Carbopol gels is prepared by dissolving 1 wt.% of Carbopol C934 in deionized water. Later, hydrogels with different concentrations were prepared by diluting the master solution.
	Vicente et al. [18]	The hydrogel is an aqueous suspension of Carbopol C934 microgels.
	Chau et al. [19]	Poly (acrylamide-*co*-acrylic acid) hydrogels were synthesized via free radical polymerization.
Guar gum with scleroglucan	Zahm et al. [20]	The hydrogel is prepared by mixing guar gum (Viscogum HV 300A, SATIA) with scleroglucan (Actigum CS 11, SATIA)
	Lafforgue et al. [21]	The hydrogel is prepared by mixing 0.5 wt. % of galactomannan with 1.5 wt. % of scleroglucan.
Guar gum with borax	Coviello et al. [22]	The hydrogel is prepared by mixing a solution of guar gum (concentration is not mentioned in the paper) with 0.1 M borax solution.
	Pan et al.[23]	The hydrogel is prepared by dissolving 0.15 g of guar gum in 10 ml of deionized water, and then 4 wt. % borax solution was added into the guar gum solution.
	Sun et al. [24]	The hydrogel is prepared by mixing 2.5 g/L guar gum solution with 2.5 g/L borax solution.

Table S2 displays the different equipment utilized by various research groups for studying the rheological moduli of mucus-mimicking hydrogels.

Table S2. Instruments used for measuring the storage and loss modulus of the hydrogels reviewed in the study.

Hydrogel	Group	Instrument to measure the rheological properties
Human airway mucus	Wolf et al. [1]	Magnetic micro rheometer
	Hill et al. [2]	Cone and plate rheometer
Poly vinyl alcohol	Krise et al. [3]	Ostwald viscometer
	Park et al. [4]	DMA 2980 dynamic mechanical analyzer
	Narita et al. [5]	Diffusing wave spectroscopy for micro rheology<br><br>Haake RS600 rheometer for macro rheology
PVA Borax	Lu et al. [7]	Rotational rheometer MARS III Haake
	Lin et al. [8]	Cone and plate geometry rheometer (plate diameter 50 mm, cone angle 0.04 rad).
	Vinod et al. [6]	Cone and plate rheometer
Polyglycerol	Sharma et al. [10]	Stress controlled MCR 501 Anton Paar rheometer with a plate-plate stainless steel geometric setup.
	Lospichl et al. [11]	Temperature controlled Bohlin Gemini 200 HR nano rheometer
	Ekinci et al. [12]	Physica MCR 501 rheometer by Anton Paar.
Polyacrylic acid	Kim et al. [15]	Parr Physica UDS 200 mechanical rheometer.
	Bonacucina et al. [16]	Stress controlled rheometer equipped with cone-plate geometry.
	Schenck et al. [17]	Haake Rheostress 1-oscillating cone and plate rheometer.
	Baek et al. [15]	Stress controlled rheometer (AR 2000, TA instrument).
	Vicente et al. [18]	Controlled strain rheometer (Rheometrics ARES-LS rheometer)<br><br>Controlled stress rheometer (Haake RS1 rheometer).
Guar gum with scleroglucan	Zahm et al. [20]	Steady shear viscoelastometer
Guar gum with borax	Coviello et al. [22]	Stress controlled rheometer (Haake Rheo stress RS 300 model).
	Sun et al. [24]	AR-G2 rheometer with cone and plate geometry.

Table S3 displays the different equipment utilized by various research groups for studying the tribological properties of mucus-mimicking hydrogels.

Table S3. Instruments used for measuring the tribological properties of hydrogels reviewed in the study

Hydrogel	Group	Tribological property measured	Instrument used
Human airway mucus	Albers et al.	Work of adhesion	Young dupre equation was used to calculate the work of adhesion. The surface tension of the hydrogel (which is plugged into the Young dupre equation to calculate the work of adhesion) is measured using du Nouy ring method.
Poly vinyl alcohol	Vinod et al. [6]	Lateral retention force	Centrifugal Adhesion Balance
PVA Borax	Cui et al. [9]	Coefficient of friction	UMT-2 tribometer
	Vinod et al. [6]	Lateral retention force and work of adhesion	Centrifugal Adhesion Balance
Polyglycerol	Le et al. [13]	Coefficient of friction and wear rate.	Thrust collar tribotester
	Orafai et al. [14]	Surface energy of the polymers	They investigated the surface energy of the polymers by measuring contact angles with different test liquids and plugging them into the Fowkes equation.
Polyacrylic acid	Chau et al. [19]	Coefficient of friction	Linear reciprocating tribometer
Guar gum with scleroglucan	Lafforgue et al. [21]	Surface tension	du Nouy ring
Guar gum with borax	Pan et al. [23]	Work of adhesion	Digital tensile machine

[1] D.P. Wolf, L. Blasco, M.A. Khan, M. Litt, Human cervical mucus. I. Rheologic characteristics, Fertil Steril. 28 (1977).

[2] D.B. Hill, B. Button, M. Rubinstein, R.C. Boucher, PHYSIOLOGY AND PATHOPHYSIOLOGY OF HUMAN AIRWAYMUCUS, Physiol Rev. 102 (2022).

[3] K.M. Krise, A.A. Hwang, D.M. Sovic, B.H. Milosavljevic, Macro- and microscale rheological properties of poly(vinyl alcohol) aqueous solutions, Journal of Physical Chemistry B. 115 (2011).

[4] J.S. Park, J.W. Park, E. Ruckenstein, On the viscoelastic properties of poly(vinyl alcohol) and chemically crosslinked poly(vinyl alcohol), J Appl Polym Sci. 82 (2001).

[5] T. Narita, K. Mayumi, G. Ducouret, P. Hébraud, Viscoelastic properties of poly(vinyl alcohol) hydrogels having permanent and transient cross-links studied by microrheology, classical rheometry, and dynamic light scattering, Macromolecules. 46 (2013).

[6] A. Vinod, Y.V. Reddy Bhimavarapu, M. Hananovitz, Y. Stern, S. Gulec, A.K. Jena, S. Yadav, E.J. Gutmark, P.K. Patra, R. Tadmor, Mucus-Inspired Tribology, a Sticky Yet Flowing Hydrogel, ACS Appl Polym Mater. (2022).

[7] B. Lu, F. Lin, X. Jiang, J. Cheng, Q. Lu, J. Song, C. Chen, B. Huang, One-Pot Assembly of Microfibrillated Cellulose Reinforced PVA-Borax Hydrogels with Self-Healing and pH-Responsive Properties, ACS Sustain Chem Eng. 5 (2017).

[8] H.L. Lin, T.L. Yu, C.H. Cheng, Reentrant behavior of poly(vinyl alcohol) - Borax semidilute aqueous solutions, Colloid Polym Sci. 278 (2000).

[9] L. Cui, J. Chen, C. Yan, D. Xiong, Mechanical and Biotribological Properties of PVA/SB Triple-Network Hydrogel for Biomimetic Artificial Cartilage, J Bionic Eng. (2022).

[10] A. Sharma, B. Thongrom, S. Bhatia, B. von Lospichl, A. Addante, S.Y. Graeber, D. Lauster, M.A. Mall, M. Gradzielski, R. Haag, Polyglycerol-Based Mucus-Inspired Hydrogels, Macromol Rapid Commun. 42 (2021).

[11] B. von Lospichl, S. Hemmati-Sadeghi, P. Dey, T. Dehne, R. Haag, M. Sittinger, J. Ringe, M. Gradzielski, Injectable hydrogels for treatment of osteoarthritis – A rheological study, Colloids Surf B Biointerfaces. 159 (2017).

[12] D. Ekinci, A.L. Sisson, A. Lendlein, Polyglycerol-based polymer network films for potential biomedical applications, J Mater Chem. 22 (2012).

[13] V.N.A. Le, J.W. Lin, Tribological properties of aluminum nanoparticles as additives in an aqueous glycerol solution, Applied Sciences (Switzerland). 7 (2017).

[14] H. Orafai, P. Kallinteri, M. Garnett, S. Huggins, G. Hutcheon, C. Pourcain, Novel poly(glycerol-adipate) polymers used for nanoparticle making: A study of surface free energy, Iranian Journal of Pharmaceutical Research. 7 (2008).

[15] G. Baek, C. Kim, Rheological properties of Carbopol containing nanoparticles, J Rheol (N Y N Y). 55 (2011).

[16] G. Bonacucina, S. Martelli, G.F. Palmieri, Rheological, mucoadhesive and release properties of Carbopol gels in hydrophilic cosolvents, Int J Pharm. 282 (2004).

[17] D.M. Schenck, J. Fiegel, Tensiometric and Phase Domain Behavior of Lung Surfactant on Mucus-like Viscoelastic Hydrogels, ACS Appl Mater Interfaces. 8 (2016).

[18] J. De Vicente, J.R. Stokes, H.A. Spikes, Soft lubrication of model hydrocolloids, in: Food Hydrocoll, 2006.

[19] A.L. Chau, P.T. Getty, A.R. Rhode, C.M. Bates, C.J. Hawker, A.A. Pitenis, Superlubricity of pH-responsive hydrogels in extreme environments, Front Chem. 10 (2022).

[20] J.M. Zahm, M. King, C. Duvivier, D. Pierrot, S. Girod, E. Puchelle, Role of simulated repetitive coughing in mucus clearance, European Respiratory Journal. (1991).

[21] O. Lafforgue, N. Bouguerra, S. Poncet, I. Seyssiecq, J. Favier, S. Elkoun, Thermo-physical properties of synthetic mucus for the study of airway clearance, J Biomed Mater Res A. 105 (2017) 3025–3033.

[22] T. Coviello, P. Matricardi, F. Alhaique, R. Farra, G. Tesei, S. Fiorentino, F. Asaro, G. Milcovich, M. Grassi, Guar gum/borax hydrogel: Rheological, low field NMR and release characterizations, Express Polym Lett. 7 (2013).

[23] X. Pan, Q. Wang, D. Ning, L. Dai, K. Liu, Y. Ni, L. Chen, L. Huang, Ultraflexible Self-Healing Guar Gum-Glycerol Hydrogel with Injectable, Antifreeze, and Strain-Sensitive Properties, ACS Biomater Sci Eng. 4 (2018).

[24] C. Sun, Y. Boluk, Rheological behavior and particle suspension capability of guar gum: sodium tetraborate decahydrate gels containing cellulose nanofibrils, Cellulose. 23 (2016).
